# Ocular biometric features of pediatric patients with fibroblast growth factor receptor-related syndromic craniosynostosis

**DOI:** 10.1038/s41598-021-85620-9

**Published:** 2021-03-17

**Authors:** Byung Joo Lee, Kihwang Lee, Seung Ah Chung, Hyun Taek Lim

**Affiliations:** 1 Department of Ophthalmology, Asan Medical Center, University of Ulsan College of Medicine, 88, Olympic-ro 43-gil, Songpa-gu, Seoul, 05505 Korea; 2 Department of Ophthalmology, Ajou University School of Medicine, 164 World Cup‑ro, Yeongtong‑gu, Suwon, 16499 South Korea

**Keywords:** Refractive errors, Paediatric research

## Abstract

Ametropia is reported as a common ophthalmic manifestation in craniosynostosis. We retrospectively compared childhood refractive error and ocular biometric features of fibroblast growth factor receptor (FGFR)-related syndromic craniosynostosis patients with those of non-syndromic craniosynostosis and control subjects. Thirty-six eyes (18 patients) with FGFR-related syndromic craniosynostosis, 76 eyes (38 patients) with non-syndromic craniosynostosis, and 114 eyes (57 patients) of intermittent exotropes were included in the analysis. Mean age at examination was 7.82 ± 2.51 (range, 4–16) years and mean spherical equivalent was -0.09 ± 1.46 Diopter. Mean age and refractive error were not different between groups, but syndromic craniosynostosis patients had significantly longer axial length, lower corneal power, and lower lens power than other groups (*p* < 0.01, *p* < 0.01, and *p* < 0.01, respectively). Axial length was positively correlated and keratometry and lens power were negatively correlated with age in non-syndromic craniosynostosis and controls, while these correlations between age and ocular biometric parameters were not present in the FGFR-related syndromic craniosynostosis. In conclusion, ocular biometric parameters in FGFR-related syndromic craniosynostosis differed from those of non-syndromic craniosynostosis and age-matched controls, and did not show the relations with age, suggesting this cohort may have abnormal refractive growth.

## Introduction

Craniosynostosis is a rare and heterogeneous disorder characterized by premature fusion of the skull plates and subsequent cranial deformities. The majority of craniosynostosis patients are isolated form (up to 85%), which is thought to be multifactorial in nature^1,2^. Other forms of craniosynostosis are genetic syndromes caused by genetic mutations in fibroblast growth factor receptors (*FGFRs*), *TWIST1*, *EFNB1*, *TCF12*, *MSX2*, or *ERF*._3_ Syndromic craniosynostosis is characterized by complex craniofacial deformities with or without malformation of the extremities and neurocognitive defects^3^. Because the incidence of syndromic craniosynostoses such as Crouzon and Apert syndromes are as low as 16.5 and 12.5 per million births, respectively^4,5^, only a few studies have evaluated the ophthalmological features of these patients.

Ophthalmic pathologies including optic atrophy, refractive error, amblyopia, and strabismus had been described in both isolated and syndromic craniosynostosis patients^6–9^. According to prior studies, hypermetropia is the most common type of ametropia in both non-syndromic and syndromic craniosynostosis^6,8–10^. However, the age at examination and definition of significant refractive error (hypermetropia, myopia, and astigmatism) differ between studies. Moreover, prior studies were not controlled for environmental factors and ethnic differences, which can affect refractive error^11–13^. Thus, the impact of syndromic and non-syndromic craniosynostosis on refractive error remains to be elucidated.

In the present study, we aimed to compare the refractive error and ocular biometric characteristics of pediatric FGFR-related syndromic craniosynostosis patients with those of non-syndromic craniosynostosis and control subjects. Furthermore, we analyzed the correlations of ocular biometric measurements with age to determine differences in the age related distribution of refractive components between the three groups.

## Results

A total of 113 patients (226 eyes) with mean age of 7.82 ± 2.51 (range, 4–16) years were included: 36 eyes from 18 FGFR-related syndromic craniosynostosis patients, 76 eyes from 38 non-syndromic craniosynostosis patients, and 114 eyes from 57 intermittent exotrope control subjects. Of the FGFR-related syndromic craniosynostosis, 14 were Crouzon Syndrome, two were Pfeiffer syndrome, one was Apert syndrome, and one was Muenke syndrome. All patients with Crouzon, Pfeiffer, and Apert syndrome carried pathogenic or likely pathogenic mutations in the *FGFR2* gene according to the 2015 American College of Medical Genetics and Genomics and Association for Molecular Pathology guidelines^14^. The patient with Muenke syndrome had a pathogenic or likely pathogenic mutation in *FGFR3*. Genetic mutations in syndromic craniosynostosis patients are summarized in Table 1.

**Table 1:** Genetic features of patients with syndromic craniosynostosis associated with fibroblast growth factor receptor mutation.

Clinical diagnosis	Gene	Variant	Number of patients	Molecular consequence	Interpretation†	Gain/loss of function
Crouzon syndrome	FGFR2	c.1061C > G (p.Ser354Cys)	2	missense	Pathogenic	Gain of function (PMID: 8,755,573)
Crouzon syndrome	FGFR2	c.1025G > A (p.Cys342Tyr)	4	missense	Pathogenic/Likely pathogenic​	Gain of function (PMID: 15,316,116)
Crouzon syndrome	FGFR2	c.1018 T > C (p.Tyr340His)	3	missense	Pathogenic	Gain of function (PMID: 8,755,573)
Crouzon syndrome	FGFR2	c.1040C > G (p.Ser347Cys)	3	missense	Pathogenic/Likely pathogenic​	Gain of function (PMID: 23,754,559)
Crouzon syndrome	FGFR2	c.1013G > A (p.Gly338Glu)	1	missense	Pathogenic/Likely pathogenic​	Undetermined
Crouzon/Pfeiffer syndrome	FGFR2	c.833G > T (p.Cys278Phe)	1 with Crouzon, 1 with Pfeiffer	missense	Pathogenic	Gain of function (PMID: 11,596,961)
Pfeiffer syndrome	FGFR2	c.1694A > C (p.Glu565Ala)	1	missense	Pathogenic/Likely pathogenic​	Gain of function (PMID: 17,803,937)
Apert syndrome	FGFR2	c.758C > G (p.Pro253Arg)	1	missense	Pathogenic	Gain of function (PMID: 9,700,203)
Muenke syndrome	FGFR3	c.749C > G (p.Pro250Arg)	1	missense	Pathogenic/Likely pathogenic​	Gain of function (PMID: 14,613,973)

FGFR, fibroblast growth factor receptor.

^†^Classification according to 2015 American College of Medical Genetics and Genomics and the Association for Molecular Pathology guidelines.

In patients with non-syndromic craniosynostosis, lamdoid suture (3/38), metopic suture (3/38), sagittal suture (12/38), coronal suture (14/38), and combined suture involvement (6/38) were observed. Cranial vault remodeling procedure was performed in all of the syndromic craniosynostosis patients and in 35 out of 38 non-syndromic craniosynostosis patients. The proportion of cranial vault remodeling surgery was not significantly different in two groups (*p* = 0.17). The mean age at cranial vault remodeling procedure in syndromic craniosynostosis and non-syndromic craniosynostosis group were 20.9 ± 24.6 and 18.1 ± 20.0 months respectively, and there was no difference in the mean age at cranial vault remodeling procedure between the two groups (*p* = 0.98). Overall, 10 out of 18 patients with syndromic craniosynostosis and 19 out of 38 patients with non-syndromic craniosynostosis patients underwent expansion cranioplasty before the first year of age. The proportion of patients who underwent expansion cranioplasty before the first year of age was not different between syndromic and non-syndromic craniosynsostosis groups (*p* = 0.78).

We’ve compared the visual acuity and presence of ophthalmic complications such as amblyopia, optic atrophy, corneal opacity and strabismus in FGFR-related syndromic craniosynostosis and non-syndromic craniosynostosis patients (Supplementary table 1). There was no significant difference in mean visual acuity in both groups (*p* = 0.94). The frequency of optic atrophy (*p* = 0.11) and amblyopia (*p* = 0.39) was also not different in each group. However, corneal opacity (*p* < 0.01) and strabismus (*p* < 0.01) were more frequently observed in syndromic craniosynostosis than in non-syndromic craniosynostosis group. In this series, all cases of corneal scarring were visually insignificant because corneal scarring in syndromic craniosynostosis only involved the inferior cornea.

**Figure 1:**
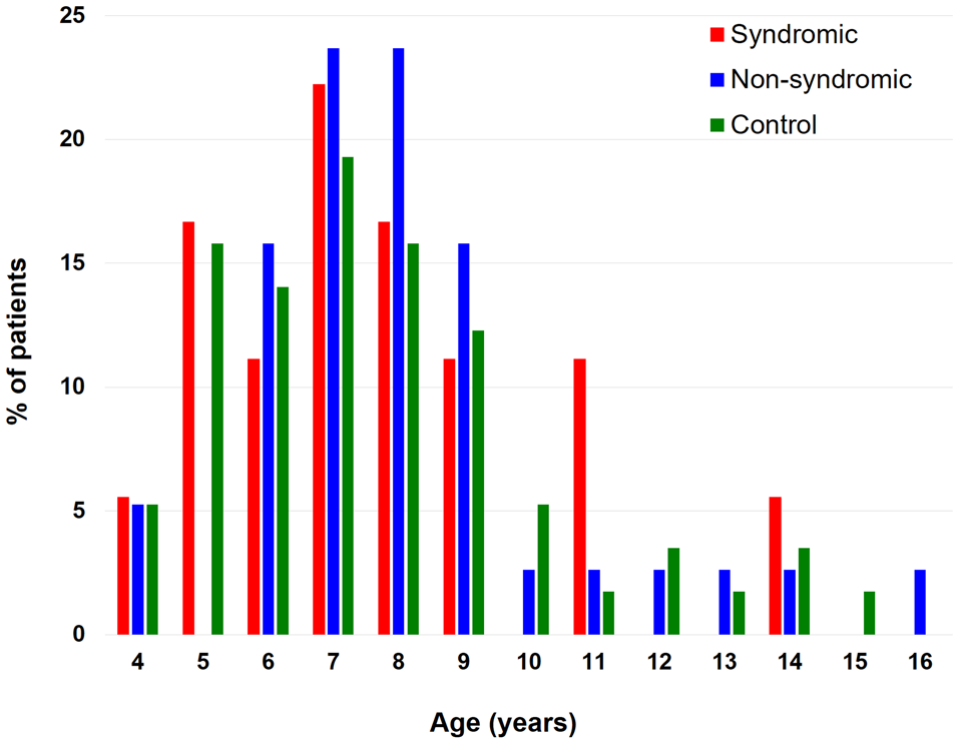
Age distribution of the syndromic craniosynostosis, non-syndromic craniosynostosis, and control groups.

**Figure 2:**
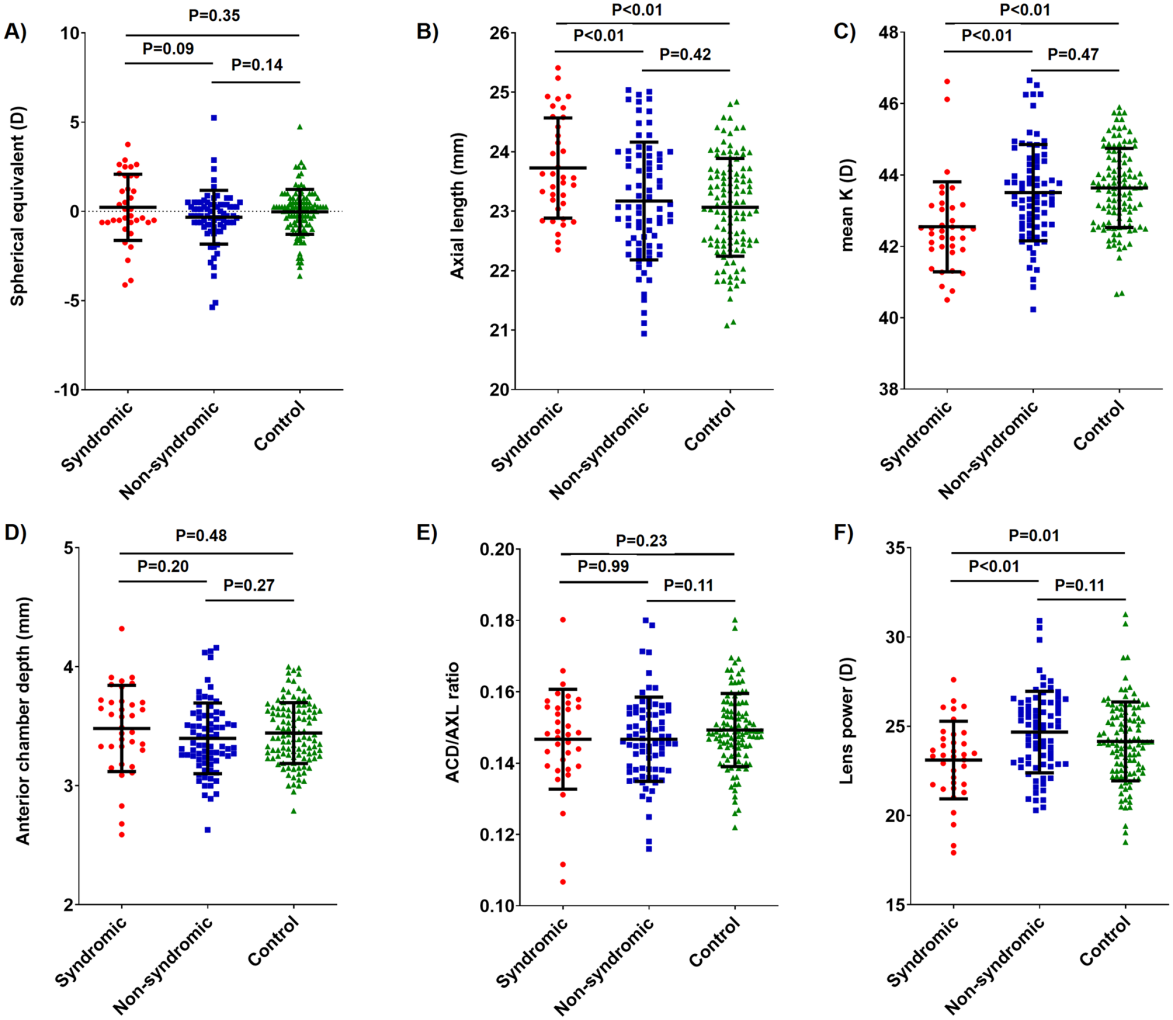
Comparison of biometric values between the syndromic craniosynostosis, non-syndromic craniosynostosis, and control groups. The distribution of (**A**) spherical equivalent, (**B**) axial length, (**C**) mean keratometric value, (**D**) anterior chamber depth, (**E**) anterior chamber depth/axial length ratio, and (**F**) lens power was compared between groups. ACD, anterior chamber depth; AXL, axial length; K, keratometry; NS, not significant. Error bars represent mean ± SD.

There was no significant difference in age at examination, sex, or mean spherical equivalent between the groups (Table 2). The age distributions of the three groups are shown in Fig. 1. The mean values of each refractive component (axial length, mean keratometry, lens power) were significantly different between the groups, while mean spherical equivalent was comparable (Table 2). Anterior chamber depth and anterior chamber depth per axial length ratio did not differ between the groups. When the head-to-head comparison of refractive error and ocular biometric values between each group was performed, mean spherical equivalent, anterior chamber depth, and anterior chamber depth/axial length ratio were not different between the groups (Fig. 2A,D,E), whereas syndromic craniosynostosis patients had longer axial length, flatter mean keratometry, and lower lens power than the other groups (Fig. 2B,C,F). There were no significant differences in mean axial length, keratometry, or lens power between the non-syndromic craniosynostosis and control group.

**Table 2:** Biometric characteristics of syndromic, non-syndromic craniosynostosis and control group.

	Syndromic craniosynostosis	Non-syndromic craniosynostosis	Control	*p* value
Eyes (patients)	36 (18)	76 (38)	114 (57)	
Age (Mean ± SD)	7.61 ± 2.49	8.09 ± 2.46	7.68 ± 2.57	0.48†
Male:Female (male ratio)	12:6 (66.7%)	21:17 (55.3%)	32:25 (56.1%)	0.69‡
SE (D)	0.23 ± 1.86	-0.33 ± 1.50	− 0.03 ± 1.26	0.14†
Mean K (D)	42.55 ± 1.26	43.50 ± 1.35	43.63 ± 1.11	< 0.01†
Lens Power (D)	23.11 ± 2.18	24.68 ± 2.28	24.15 ± 2.21	< 0.01†
AXL (mm)	23.73 ± 0.84	23.17 ± 0.99	23.07 ± 0.82	< 0.01†
ACD (mm)	3.48 ± 0.36	3.40 ± 0.30	3.44 ± 0.26	0.33†
ACD/AXL ratio	0.15 ± 0.01	0.15 ± 0.01	0.15 ± 0.01	0.24†

ACD, anterior chamber depth; AXL, axial length; K, keratometry; SD, standard deviation; SE, spherical equivalent; †, one-way ANOVA test; ‡, Fisher’s exact test.

When we performed subgroup analysis for biometric values of the patients who underwent cranial vault remodeling procedures, axial length was significantly longer in syndromic craniosynostosis (23.73 ± 0.84 mm) than that in non-syndromic group (23.17 ± 0.96 mm; *p* < 0.01). Mean keratometric value was significantly smaller in syndromic craniosynostosis patients (42.55 ± 1.26 Diopter) than that in non-syndromic ones (43.44 ± 1.33 Diopter; *p* < 0.01) and lens power was also lower in syndromic group (23.11 ± 2.18 Diopter) than in non-syndromic group (24.75 ± 2.25 Diopter; *p* < 0.01). Then we performed a subgroup analysis according to the age at cranial vault remodeling procedures: early surgery group (before 1st year of age; n = 29) versus late surgery group (after 1st year of age; n = 24). In early surgery group, mean keratometric value (*p* = 0.02) and lens power (*p* < 0.01) was still significantly lower in syndromic craniosynostosis patients than in non-syndromic group. However, mean value of axial length was not significantly different between the syndromic and non-syndromic craniosynostosis patients (*p* = 0.14). In late surgery group, axial length was significantly longer (*p* = 0.02) and mean keratometric value was significantly lower (*p* < 0.01) in syndromic craniosynostosis patients than in non-syndromic group. But, mean keratometric value was not significantly different between the syndromic and non-syndromic craniosynostosis patients (*p* = 0.13).

When patients were subgrouped into younger (4–8 years) and older (≥ 9 years) age groups, mean axial length was significantly longer in the young syndromic craniosynostosis subgroup (23.79 ± 0.88 mm) relative to the young subgroups of non-syndromic craniosynostosis (22.87 ± 0.89 mm; *p* < 0.01) and control (22.91 ± 0.74 mm; *p* < 0.01), while there was no significant difference between the mean value of axial length from young subgroups of non-syndromic craniosynostosis and control subject (*p* = 0.66). In contrast, axial length did not differ between the groups in older patients. The mean axial length in older subgroup of syndromic craniosynostosis, non-syndromic craniosynostosis, and control subjects were 23.57 ± 0.75 mm, 23.86 ± 0.88 mm, and 23.44 ± 0.90 mm, respectively (*p* = 0.20, one-way ANOVA). In younger subgroup, mean keratometric value and mean lens power were significantly lower in syndromic craniosynostosis patients than that in non-syndromic craniosynostosis (*p* < 0.01 for keratometry; *p* < 0.01 for lens power) and control (*p* < 0.01 for keratometry; *p* = 0.05 for lens power). Contrastingly, in older subgroup, there was no significant difference between the mean keratometric value and mean lens power of syndromic, non-syndromic craniosynostosis, and control subjects (*p* = 0.90 for keratometry; *p* = 0.23 for lens power, one-way ANOVA).

**Figure 3:**
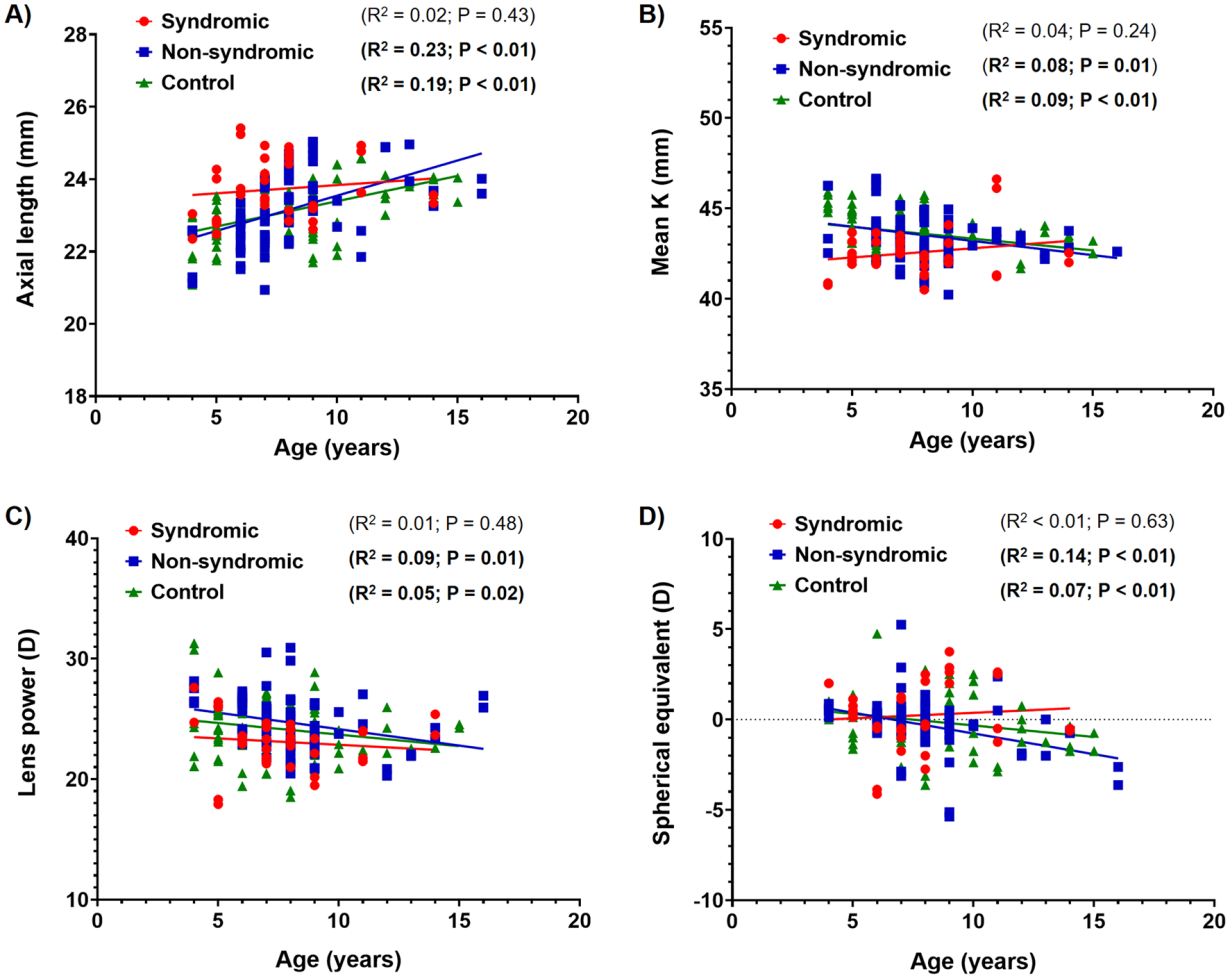
Scatter plots of biometric values and refractive errors according to age. Linear regression analysis between age and (**A**) axial length, (**B**) mean keratometry, (**C**) lens power, and (**D**) spherical equivalent in syndromic craniosynostosis, non-syndromic craniosynostosis, and control groups. K, keratometry.

To delineate the differences in age-specific distribution of refractive error and ocular biometric values from the syndromic craniosynostosis, non-syndromic craniosynostosis, and control groups, linear regression analysis was performed (Fig. 3). Spherical equivalent refractive error was negatively correlated with age in non-syndromic craniosynostosis [spherical equivalent = -0.23*Age + 1.53 (R^2^ = 0.14; *p* < 0.01)] and control subjects [spherical equivalent = -0.13*Age + 0.95 (R^2^ = 0.07; *p* = 0.01)]. However, no such correlation was detected in syndromic craniosynostosis patients [spherical equivalent = 0.06*Age – 0.24 (R^2^ = 0.01; *p* = 0.63)]. The distribution of axial length was positively correlated with age in the non-syndromic craniosynostosis [axial length =  + 0.19*Age + 21.60 (R^2^ = 0.23; *p* < 0.01)] and control group [axial length =  + 0.14*Age + 21.99 (R^2^ = 0.19; *p* < 0.01)]. However, no significant age-axial length correlation was detected in syndromic craniosynostosis patients [axial length = 0.05*Age + 23.38 (R^2^ = 0.02; *p* = 0.43)]. Age and mean keratometric value were negatively correlated in the non-syndromic craniosynostosis [mean keratometry = -0.16*Age + 44.78 (R^2^ = 0.08; *p* = 0.01)] and control groups [mean keratometry = -0.13*Age + 44.64 (R^2^ = 0.09; *p* < 0.01)], while no significant age-related decrease of keratometric value was noted in the syndromic craniosynostosis group [mean keratometry =  + 0.10*Age + 41.77 (R^2^ = 0.04; *p* = 0.24)]. Age and lens power were negatively correlated in non-syndromic craniosynostosis [lens power = -0.27*Age + 26.89 (R^2^ = 0.09; *p* = 0.01)] and control subjects [lens power = -0.19*Age + 25.63 (R^2^ = 0.05; *p* = 0.02)]. In contrast, age and lens power were not correlated in syndromic craniosynostosis subjects [lens power = -0.11*Age + 23.92 (R^2^ = 0.01; *p* = 0.48)].

## Discussion

Ametropia is consistently suggested as one of the major ophthalmic manifestations of both syndromic and non-syndromic craniosynostosis, but age and ethnic group-controlled comparative studies evaluating the refractive errors of syndromic and non-syndromic craniosynostosis patients have not reported yet^6,8–10^. To the best of our knowledge, this is the first comparative study of refractive errors in FGFR-related syndromic and non-syndromic craniosynostosis. In the present study, we demonstrated that pediatric FGFR-related syndromic craniosynostosis is characterized by longer axial length, lower mean keratometric value, and lower lens power than the non-syndromic craniosynostosis and control groups, while mean refractive error was not significantly different between the groups.

Mean anterior chamber depth and anterior chamber depth/axial length ratio were not significantly different between the groups. Taken together, the lower refractive index of the cornea and lens dampen the potential myopic defocus due to longer axial length in syndromic craniosynostosis.

Age-dependent myopic shift was not prominent in syndromic craniosynostosis patients compared to non-syndromic craniosynostosis and control subjects. In terms of age-related distribution of ocular biometric components, the non-syndromic craniosynostosis and control groups exhibited age-dependent axial elongation as previously reported^15,16^. Although syndromic craniosynostosis patients had longer mean axial length, this age-related trend was not observed in the syndromic craniosynostosis group. Moreover, inter-group axial length difference was only significant in the younger (4–8 years) subgroup. During longitudinal axial growth, rapid axial growth occurs before 5 years of age, and a slower growth phase then ensues until 13 years of age^17^. Therefore, FGFR-related syndromic craniosynostosis patients could have had a more prominent early axial growth spurt than non-syndromic craniosynostosis and control groups.

Loss of cornea and lens power in school-age children has been well documented by several longitudinal studies^16,18,19^. Age-related elongation of axial length and accompanying deterioration of corneal and lens power could be explained by emmetropization. According to our study, unlike the non-syndromic craniosynostosis and control groups, the age-related deterioration of keratometric value and time-dependent loss of lens power were not noted in the syndromic craniosynostosis group. Therefore, the authors have assumed that the emmetropization mechanism is involved in the stable axial length and corneal/lens refractive indices observed in syndromic craniosynostosis subjects.

*FGFR*s (*FGFR1-3*), *TWIST*, and *MSX2* gene mutations are the major causes of syndromic craniosynostosis. Common genetic syndromes associated with craniosynostosis, including Crouzon-Pfeiffer syndrome, Apert syndrome, and Muenke syndrome, are caused by pathogenic mutations in *FGFR*s. Most of the pathogenic *FGFR* mutations in syndromic craniosynostosis are gain-of-function mutations^10,20,21^. Fibroblast growth factors (FGFs) are a family of growth factors that exert diverse physiological roles via FGFRs. FGFs are essential factors for ocular development during both prenatal and postnatal periods. FGFRs are well known to be expressed in the mammalian and avian sclera^22,23^. Thus, FGFs are thought to be involved in the axial growth of ocular dimensions. However, there are contradictory results on whether excessive FGFs could induce axial refractive growth^24,25^. FGFs are also known as crucial factors for lens epithelial growth and differentiation^26–28^, but the role of FGFs in refractive growth of the crystalline lens is largely unknown. In the present study, all of the syndromic craniosynostosis subjects were genetically proven to have pathological or likely pathological mutations of *FGFR2/3*. Considering that non-syndromic craniosynostosis patients shared the same refractive growth pattern as control patients, the unique ocular biometric characteristics of FGFR-related syndromic craniosynostosis could be at least partly attributed to overactivation of FGFRs.

There are some limitations of the study that should be acknowledged to avoid overinterpretation of the findings. First, due to the cross-sectional nature of the study, a head-to-head comparison of time dependent refractive growth patterns between study groups could not be performed. A longitudinal study analyzing the time-dependent change of refractive error and ocular biometric values should be conducted to confirm the characteristic refractive growth pattern of syndromic craniosynostosis patients suggested by the present study. Second, FGFR-related syndromic craniosynostosis is a rare disease entity and is often accompanied by cognitive dysfunction, which is an obstacle for measurement of ocular biometric values. Therefore, fewer syndromic craniosynostosis subjects were available for analysis than non-syndromic craniosynostosis and control subjects.

In conclusion, although mean refractive error of pediatric FGFR-related syndromic craniosynostosis patients did not differ from that of non-syndromic craniosynostosis and control subjects, pediatric FGFR-related syndromic craniosynostosis patients had longer axial length, flatter keratometry, and lower crystalline lens power than other two groups. Age-associated distribution of refractive error and ocular biometric values in pediatric FGFR-related syndromic craniosynostosis patients was also strikingly different from that of non-syndromic craniosynostosis and control subjects. Early axial elongation and stable biometric values throughout adolescence can be characteristic of pediatric FGFR-related syndromic craniosynostosis patients.

## Methods

We retrospectively reviewed the medical records of craniosynostosis patients who were referred to a pediatric ophthalmology clinic of Ajou university hospital for ophthalmological evaluation between March 2019 and February 2020. The institutional review board (IRB) of Ajou university hospital reviewed and approved the study (IRB approval number: MED-MDB-20–399). This study was performed in accordance with institutional guidelines of Ajou University Hospital and Informed consent was waived by the IRB of Ajou university hospital because of the retrospective nature of the study.

The diagnosis of craniosynostosis was made at the Department of Medical Genetics and Department of Pediatric Neurosurgery in Ajou university hospital after detailed clinical, radiological examination, and genetic evaluation. Systemic abnormalities such as maxillofacial dysmorphism, ear anomaly, cleft lip and palate, hearing problem and anomalies of the phalanges were carefully checked by medical geneticists to rule out syndromic craniosynostosis. Sanger sequencing analysis of *FGFR1*, *FGFR2*, and *FGFR3* hot spot mutations was performed for genetic diagnosis of FGFR-related syndromic craniosynostosis. In patients with craniosynostosis, a comprehensive ocular examination, including ocular alignment, refractive errors, biometric measurements, slit-lamp examination, and fundus examination, were performed. Refractive errors were measured by cycloplegic refraction and ocular biometry (axial length, keratometric value, and anterior chamber depth) using a partial coherence interferometry, IOL Master 500 device (Carl Zeiss Meditec AG, Jena, Germany). During the study period, cycloplegic refraction and ocular biometric measurements were routinely performed in all new patients that were diagnosed with intermittent exotropia at the pediatric ophthalmology clinic. These patients with intermittent exotropia were served as non-craniosynostosis controls.

Craniosynostosis patients and intermittent exotropia patients that underwent measurement of refractive error under cycloplegia and ocular biometry during the study period were included. Clinical information, including diagnosis, history of systemic and ocular disease, gender, age, refractive error, axial length, keratometric value, and anterior chamber depth, were collected. We’ve reviewed the visual acuity and presence of ophthalmic complications such as amblyopia, optic atrophy, corneal opacity and strabismus in FGFR-related syndromic craniosynostosis and non-syndromic craniosynostosis patients. For patients with FGFR-related syndromic craniosynostosis, genetic information was also collected. Patients with a history of systemic disease other than craniosynostosis syndrome or with a history of ocular disease other than intermittent exotropia were excluded. Patients in which biometric values were not measured due to poor cooperation or with low signal-to-noise ratio values (less than 2.1) in IOL master measurement were also excluded.

Crystalline lens power was estimated based on refractive error and ocular biometric values using Bennett–Rabbetts methods^29^. We comprehensively reviewed the literature and ClinVar database (https://www.ncbi.nlm.nih.gov/clinvar/) to determine the functional consequences of the *FGFR* mutations detected in each patient.

Categorical clinical characteristics of syndromic craniosynostosis, non-syndromic craniosynostosis, and control eyes were compared by chi-square test. Mean value of refractive errors and biometric measurements from each groups compared by Mann–Whitney U test. The mean value of each variable was represented as mean ± standard deviation. A one-way ANOVA test assuming unequal variances was performed to compare the rates of refractive growth between the three groups. Linear regression was used to determine if the rate of refractive growth was correlated with changes in biometric values in the three groups.

## Supplementary Information

Supplementary Information
