# Supplementary material for: Ocular biometric features of pediatric patients with fibroblast growth factor receptor-related syndromic craniosynostosis
Source: Sci Rep. 2021 Mar 17;11:6172. doi: 10.1038/s41598-021-85620-9 (PMC7969619; doi:10.1038/s41598-021-85620-9)
Supplement: Supplementary file 1 — Supplementary Information [file 41598_2021_85620_MOESM1_ESM.docx]

**Title:** Ocular biometric features of pediatric patients with fibroblast growth factor receptor-related syndromic craniosynostosis

**Authors:** Byung Joo Lee^1^, Kihwang Lee^2^, Seung Ah Chung^2*^, Hyun Taek Lim^1*^

^1^Department of Ophthalmology, Asan Medical Center, University of Ulsan College of Medicine, Seoul, Korea

^2^Department of Ophthalmology, Ajou University School of Medicine, Suwon, South Korea

***Correspondence and reprint requests to:**

Seung Ah Chung, MD

Department of Ophthalmology, Ajou University School of Medicine, 164 World Cup‑ro, Yeongtong‑gu, Suwon 16499, South Korea

Tel: 82-31-219-7814, Fax: +82-31-219-5259, E-mail: mingming8@naver.com

Hyun Taek Lim, MD

Department of Ophthalmology, Asan Medical Center, University of Ulsan College of Medicine, 88, Olympic-ro 43-gil, Songpa-gu, Seoul 05505, Korea, Tel: 82-2-3010-3672, Fax: 82-2-470-6440, E-mail: htlim@amc.seoul.kr

Supplementary table 1. The ophthalmic features of FGFR-related syndromic and non-syndromic craniosynostosis

|  | **FGFR-related syndromic craniosynostosis** | **Non-syndromic craniosynostosis** | ***p*-value** |
| --- | --- | --- | --- |
| Eyes (patients) | 36 (18) | 76 (38) |  |
| Visual acuity (LogMAR) | 0.10 ± 0.24 | 0.06 ± 0.10 | 0.94† |
| Amblyopia (eyes) | 7 (19.4%) | 9 (11.8%) | 0.39‡ |
| Corneal opacity (eyes) | 5 (13.9%) | 0 (0%) | <0.01‡ |
| Optic atrophy (eyes) | 7 (19.4%) | 6 (7.9%) | 0.11‡ |
| Strabismus (patients) | 17 (94.4%) | 9 (23.7%) | <0.01‡ |

†, Mann-Whitney U test; ‡, Fisher’s exact test
